# Sudden Cardiac Death in Schizophrenia During Hospitalization: An Autopsy-Based Study

**DOI:** 10.3389/fpsyt.2022.933025

**Published:** 2022-07-01

**Authors:** Yuanyuan Chen, Fu Zhang, Yanan Yan, Shiquan Wang, Le Zhang, Fengping Yan

**Affiliations:** ^1^Department of Forensic Medicine, School of Basic Medical Sciences, Gannan Medical University, Ganzhou, China; ^2^Key Laboratory of Prevention and Treatment of Cardiovascular and Cerebrovascular Disease of Ministry of Education, Gannan Medical University, Ganzhou, China; ^3^Criminal Technology Center of Guangdong Province Public Security Bureau, Guangzhou, China; ^4^Forensic Center of Gannan Medical University, Ganzhou, China

**Keywords:** schizophrenia, hospitalization, sudden cardiac death, antipsychotic, autopsy

## Abstract

Schizophrenia is a severe mental disorder that is often comorbid with heart dysfunction and even sudden cardiac death (SCD). Clinical studies of SCD in schizophrenia have been largely reported, while there are limited autopsy studies that directly showed whole-scale information of such events. In this study, we present nine autopsy-based SCD cases in schizophrenia patients who died suddenly during hospitalization. Their medical records before and during hospitalization, and postmortem autopsy findings were summarized. These decedents had an average duration of schizophrenia for 6.83 ± 3.75 years with a male/female ratio of 4:5. They were all on intermittent antipsychotics medication before hospitalization and died within 15 days after hospitalization. Seven of the nine cases (77.8%) died of organic heart diseases such as severe coronary artery atherosclerosis (*n* = 4), myocarditis (*n* = 1), cardiomyopathy (*n* = 1), and pulmonary thromboembolism (*n* = 1). Two cases remained unexplained after systemic autopsy and toxicological examinations. Postmortem autopsy identified hepatic steatosis (*n* = 6) and respiratory inflammation (*n* = 3) as the most common associate extra-cardiac lesions. Our data provided autopsy-based data of SCD cases in schizophrenia and highlighted an intensive care of such patients during hospitalization.

## Introduction

Schizophrenia is a debilitating mental illness with a range of positive symptoms such as delusions, hallucinations, and negative symptoms such as amotivation and social withdrawal (1). The disorder is also associated with cognitive symptoms such as defects in working memory, executive function and processing speed (2). Currently, diagnosis of schizophrenia is not a challenging issue. The Diagnostic and Statistical Manual (DSM) classification system for Mental Disorders, built by American Psychiatric Association, has been employed as the diagnostic guideline in the Western Countries for years (2), while in China, the Chinese Criteria for Classification and Diagnosis of Mental Disorder version 3.0 (CCMD-3) which is minor modified from the DSM classification system, has been applied in clinic. However, major concerns that frustrate the society are not the diagnosis of this disorder but rather the high incidence of sudden deaths in this particular cohort. Based on a statistic, the life expectancy in patients with schizophrenia is ~10–25 years shorter than of the general population (3). Well known reasons for these deaths are related to unnatural death including suicides, accidents, violence, and substance abuse. A substantial proportion of individuals with schizophrenia die of natural cause such as cardiovascular diseases, followed by cancer, diabetes and pulmonary disease (4). SCD is typically defined as death from a cardiac (cardiovascular) cause within a short time (minutes to hours) after symptom initially appears, often without warning (5). The incidence of SCD in patients with schizophrenia is about 4 times higher than in the general population. The majority of SCD in schizophrenia patients are related to ischemic or structural heart diseases, including coronary artery disease, myocardial infarction, myocarditis. A small portion of the sudden death remains unexplained, which are presumed to be due to cardiac arrhythmias (6, 7). Unhealthy life-style, antipsychotics mediation and genetic factors are indicated to contribute to the excessive SCD among schizophrenia (8).

The phenomenon of SCD in this particular population has received a special attention of the scientific community. Basic and clinical research has been providing increasingly detailed information about the etiology, diagnosis, classification and treatment of schizophrenia. However, there are relatively limited autopsy data on causes of SCD in patients with schizophrenia especially in the patients during hospitalization. In this study, we present 9 patients who had been clearly diagnosed of schizophrenia with antipsychotic mediation, died suddenly and unexpectedly during hospitalization. Their clinical and forensic characteristics were summarized. The key pathological findings and final causes of death were discussed. We aim to provide further insights into schizophrenia patients suffering from SCD during hospitalization in a forensic perspective.

## Materials and Methods

### Case Collection

Cases were collected between January 2011 and December 2020 in the Forensic Center of Gannan Medical University. The inclusion criteria were:

- All the cases were clearly diagnosed of schizophrenia clinically.
- All the patients died suddenly and unexpectedly within 1 h of new symptom onset during hospitalization.
- Systematic postmortem examination was made including detailed macroscopic and microscopic examination by qualified forensic pathologists. Toxicological test was routinely conducted and the results were accessible for the cases.
- Causes of death for these patients were made without controversy by three independent pathologists. In case of multiple pathological changes, the severity of each pathological change and its contribution to the death was seriously evaluated and independently decided by the three pathologists. In cases with inconsistent conclusion, this case was consulted by another external pathologist to reach the final decision.

All patients were clearly diagnosed of schizophrenia in clinic based on the Chinese Criteria for Classification and Diagnosis of Mental Disorder version 3.0 (CCMD-3). For the standard toxicological screening, a two-step analysis was routinely performed, namely an initial qualitative analysis using gas chromatography, and a second quantitative analysis using liquid chromatography tandem mass spectrometry. Procedures for quantitative analysis of suspected substances were in accordance with the Occupation Standards for Detection of Poisons in Blood and Urine (SF/Z JD 0107005-2010 and SF/Z JD 0107005-2016). Patients who suffer from suicide, accidents, and violence were excluded. Patients dying from drug intoxication, as revealed by postmortem toxicological analysis, were excluded.

### Data Extraction

Medical records of the individual cases include demographic information, duration of psychiatric disorders, lifestyle, length of hospital stay prior to death, medication regimen during hospitalization.

Forensic records in each case include the external examination, pathological features of all organs including macro and micro findings. The forensic records also include standard toxicological screening results.

Each case was anonymized to protected patient's privacy. This study only extracted patient's information from archived records without using patients' specimens. Review of patients' medical and forensic records was approved by the Ethical Review Board at the School of Basic Medical Science, Gannan Medical University (Approval No.: 2021-217).

## Results

### Patients' Basic Characteristics Before Hospitalization

A total of 12 cases with a clear medical history of schizophrenia were referred to our center for autopsy during the study period. Among which nine cases (75.0%) were identified to die from SCD during hospitalization, while the other three cases each died from choking, falling from height, and hanging. Basic characteristics of the nine cases died from SCD were summarized in Table 1.

**Table 1:** Clinical and demographic information of the patients (*n* = 9).

**Case no**	**Age**	**Gender**	**Duration of disease**	**Life-style factor**	**Symptoms at admission**	**In hospital regimen**	**Drug dose**	**Hospitalization days**
1	45y	Male	10y	Smoking, drinking,	Odd behaviors, deterioration of self-care abilities for 4 days.	Risperidone	2 mg/day	15 days (in psychiatric department of local general hospital)
2	38y	Female	4y	Obesity, BMI 37.4	Disorganized speech, violent behavior for 1 week.	Risperidone	4 mg/day	12 days (in psychiatric department of local general hospital)
3	33y	Male	6y	None	Disinterest in the environment, refusal to speak for 4 days.	Clozapine	50 mg/day	3 days (in psychiatric department of local general hospital)
4	53y	Female	7y	None	Disorganized speech, odd behaviors, suspiciousness for 3 days	Clozapine	20 mg/day	5 days (in tertiary specialized psychiatric hospital)
5	43y	Female	15y	Obesity, BMI 30.4	Odd behaviors, lack enjoyment in daily activities, be difficulty in functioning normally for 1 week.	Clozapine, risperidone	25 mg/day, 2 mg/day respectively	5 days (in tertiary specialized psychiatry hospital)
6	46y	Male	5y	Smoking, drinking	Disorganized speech, self-inflicted violence for 1 day.	Risperidone	2–6 mg/day	2 days (in tertiary specialized psychiatry hospital)
7	52y	Female	10y	None	Irritability, odd behaviors for 3 days.	Risperidone	2 mg/day	4 days (in psychiatric department of local general hospital)
8	47y	female	2y	None	Disorganized speech, odd behaviors for 1 week.	Risperidone	4 mg/day	32 h (in psychiatric department of local general hospital)
9	30y	Male	2.5y	Smoking	Aggressive behavior for 1 h.	None	None	30 min (in local general hospital)

The nine patients comprised of four males (44.4%) and five females (55.6%), with the age ranging from 30 to 53 years (43.0 ± 7.1 years). All the patients were rigorously diagnosed with schizophrenia in clinic with the disease duration ranging from 2 to 15 years (6.83 ± 3.75 years). Two female patients presented obesity (BMI = 30.4 and 34.7, respectively). Three male patients have a smoking history, two of whom additionally had drinking history. Other patients were not recorded of unwelcome lifestyles. By recording their medical history, all the patients received medical treatments intermittently when psychiatric symptoms occurred and received antipsychotic regimens in a therapeutic dose range. They withdraw drugs when symptoms improved. None of the patients had a record of cardiac diseases before hospitalization.

### Patients' Clinical Characteristics During Hospitalization

Among the nine patients, eight of them were hospitalized with obvious symptoms. Duration of these symptoms ranges from 1 to 7 days before hospitalization. They all died suddenly and unexpectedly in daytime during hospitalization with the survival time <1 h. The length of the last hospital stay ranged from 32 to 12 d (5.94 ± 4.95 days). The death events occurred either in a psychiatry department of a general hospital or in a tertiary specialized psychiatry hospital. All patients received regular antipsychotic drug regimen during hospitalization. The 9th patient was restrained by policemen and carried to hospital for his typical aggressive behaviors occurring in a morning. Mono-therapy was more prevalent such as Risperidone in five patients, Clozapine in two patients. Poly-pharmacy was recorded in one patient. Drugs were taken all by oral administration in a therapeutic dose. The 9th patient died suddenly and shortly after arrival at hospital and did not receive any specific antipsychotic treatment, the whole hospitalization of whom lasted about 30 min.

### Autopsy Findings

Autopsy findings were summarized in Table 2. Among the nine patients, organic heart disease were found in seven patients (77.8%), including severe coronary arteriosclerosis (four cases, 57.1%), dilated cardiomyopathy (one case, 14.3%), acute myocarditis (one case, 14.3%), pulmonary thromboembolism (one case, 14.3%). In two patients none severe alterations of the heart and other solid organs were found (the so-called negative autopsy). All the nine patients showed general signs of sudden death with multiple organ congestion, particularly acute pulmonary congestion. Associate pathology changes in other organs include hepatic steatosis in six patients, bronchopneumonia in two patients, and chronic interstitial pneumonia in one patient. Antipsychotic medications were tested positive in postmortem heart blood but all were in their therapeutic levels.

**Table 2:** Autopsy findings of the deceased patients (*n* = 9).

**Case no**.	**Heart weight, g**	**LVT cm**	**RVT, cm**	**Cardiovascular pathology**	**Associate pathology in other organs**	**Cause of death**
1	265	1.2	0.2	No specific pathological alterations	Hepatic steatosis; multiple organ congestion	Cardiac arrhythmia
2	435	1.3	0.3	Atherosclerosis of the LAD (>75% luminal narrowing) and RCA (25% luminal narrowing) with fresh/old infarction	Hepatic steatosis; bronchopneumonia; multiple organ congestion	Coronary artery atherosclerosis
3	400	1.3	0.3	LV cavity dilation with myocyte hypertrophy and interstitial fibrosis	Multiple organ congestion	Dilated cardiomyopathy
4	275	1.0	0.2	Atherosclerosis of the LAD (>75% luminal narrowing) with plaque disruption and fresh/old infarction.	Hepatic steatosis; multiple organ congestion	Coronary artery atherosclerosis
5	320	1.3	0.3	Bilateral pulmonary thromboembolism	Multiple organ congestion	Pulmonary thromboembolism
6	390	1.3	0.3	Atherosclerosis of the LAD (>75% luminal narrowing) with plaque disruption and fresh/old infarction.	Hepatic steatosis; chronic pulmonary inflammation; multiple organ congestion	Coronary artery atherosclerosis
7	315	1.1	0.2	Interstitial inflammatory infiltrate and focal necrosis of the myocardium	Hepatic steatosis; bronchopneumonia; multiple organ congestion.	Acute myocarditis
8	315	1.2	0.3	No specific pathological alterations	Multiple organ congestion.	Cardiac arrhythmia
9	460	1.3	0.3	Atherosclerosis of the LAD (>50% luminal narrowing) with coronary thrombosis and fresh infarction.	Hepatic steatosis; multiple organ congestion; kidney stone	Coronary artery atherosclerosis

## Discussion

Schizophrenia is associated with a nearly 20-year reduced life expectancy compared with the general population (3). Reasons for this excess mortality are multifactorial, but cardiovascular disease that often resulted in SCD represented the leading cause of death (9, 10). Unhealthy lifestyle, such as smoking, substance use, an unbalanced diet, and reduced physical activities could ultimately lead to increased risks of SCD (11, 12). Adverse effects of antipsychotics may further result in cardiovascular risk. Chronic exposure to antipsychotics may directly damage the cardiac muscles leading to an irreversible impairment, mostly by prolongation of the QT interval, blocking potassium channel, which in turn result in life-threatening ventricular arrhythmias (13, 14). The adverse effects of antipsychotic occurred in patients with ischemia heart disease or cardiomyopathy as well as structurally normal hearts, and occurred mostly after withdrawal or discontinuation of potentially successfully regimens (15, 16). Genetic background may also predispose schizophrenia patients to SCD, while the link between genetics and the incidence of SCD among schizophrenia remain unclear (17).

Studies based on autopsy in patients with schizophrenia and SCD have been scarcely reported in the past (18–20), only one autopsy study had reported the sudden unexpected death in schizophrenia during hospitalization (18). In our study, 12 cases died suddenly during hospitalization, among which nine cases (75.0%) died from SCD. This proportion is basically consistent with a previous report showing that 78.9% of schizophrenia patients died from SCD. The specific causes of SCD included severe coronary arteriosclerosis (*n* = 4), dilated cardiomyopathy (*n* = 1), acute myocarditis (*n* = 1), pulmonary thromboembolism (*n* = 1). The remaining two cases were not revealed of positive pathological changes, the cause of whose death was presumably to be cardiac arrhythmia. No case died from drug intoxication or anaphylactic shock associated with the antipsychotic use. Comparing with those early studies, the decedents in our study were significantly younger with shorter disease duration. In particular, our case series died within 15 days after hospitalization and died without any warning even in specialized psychiatry hospitals. Therefore, our study represents the first autopsy-based report that highlighted intensive monitoring of these patients particularly within 15 days after hospitalization.

By analyzing their medical data, many factors may contribute to the SCD events in younger patients with relatively short hospitalization duration: (1) Unhealthy life-style and the related cardiovascular risk factors like obesity, diabetes, and dyslipidemia. In our patients, three male patients had a drinking history, two of whom were additional smokers. Two female patient presented obesity (BMI = 30.4 and 34.7, respectively). (2) Cardiac side effects of antipsychotics and neglect of the ECG monitoring. All our patients had received antipsychotic drugs before last hospitalization but suddenly withdrawn when symptoms being improved. During hospitalization, Risperidone was prescribed in five patients, and Clozapine was prescribed in two patients. Poly-pharmacy was recorded in one patient. For all the patients, six were not receiving regular ECG monitoring during hospitalization. (3) Socioeconomic factors. All our nine patients were from remote mountain area, without a high quality of health care system. They rarely followed screening programs or attend regular health examinations. Our study, together with those previous autopsy studies, implied that the recognition and correction of the risk factors, e.g., abstinence from smoking, exercise, weight reduction, control of blood glucose and lipid abnormalities, would likely reduce the total mortality as well as the incidence of SCD. From the clinical perspective, a routine assessment with an ECG for patients receiving antipsychotic medications may become a priority for the psychiatrists to avoid fatal outcomes. Improving the health care system and availability of clinical and community service for the schizophrenia patients may reduce the incidence of SCD.

Genetic background may also predispose schizophrenia patients to SCD. Huertas V et al. found strong evidence that a common missense variant in the neuregulin 1 gene is associated with both schizophrenia and sudden cardiac death (21). Christiansen et al. found that heart-related disease gene variants are not overrepresented among deceased individuals with schizophrenia, but the overall polygenetic burden of variants in the investigated heart genes is higher in individuals with schizophrenia compared to the general population (22). In our cases, whether they had a heredity heart disease were not recorded in their medical records, and the postmortem genetic analysis were not performed, thus we were unable to analyze the presence of genetic variation among these patients. We will be cordially interested to do postmortem genetic tests for such cases in the future.

Schizophrenia is increasingly recognized as a systemic disorder with an additional burden in terms of somatic disorders, characterized by elevated cortisol levels, dysfunction of the autonomic nervous system, inflammation, lipid abnormalities, oxidative stress and increased platelet reactivity, all of which contribute to the development and progress of cardiovascular disease and SCD (23). After systematic postmortem examinations, we found that hepatic steatosis was presented in six patients, bronchopneumonia in two patients, and chronic pulmonary inflammation in one patient. Hepatic stenosis is considered to be the hepatic manifestation of metabolic syndromes (MetS). MetS is defined by combination of abdominal obesity, high blood pressure, low high-density lipoprotein cholesterol, elevated triglycerides, and hyperglycemia (24). MetS was long considered to be a comorbid outcome of schizophrenia after years of medication even in young patients, associating with the increasing risk for SCD and CVD mortality among schizophrenia (25, 26). Clinical studies suggested that the incidence of hepatic steatosis may serve as a predictor of CVD both in the general population and in patients with schizophrenia (27). Inflammation had been hypothesized as a potential mechanism linking the immune response to the pathogenesis of schizophrenia. Several cytokines such as interleukin 1β, interleukin 6, and C-reactive protein were higher in individuals with schizophrenia (28). Infectious disease after long-term anti-psychotic use in patients with psychiatric illness had often been over-looked (29). In our patients, the high incidences of hepatic steatosis and respiratory inflammation mirror the prevalence of MetS and inflammation in schizophrenia, and therefore may explain the premature death occurred with shorter disease duration. Thus, in a clinical perspective, simultaneous treating psychiatric disorders and MetS and systemic inflammation is highly appreciated in order to enhance treatment outcomes in these patients.

In schizophrenia, some cases may have no definitive cause of death even after systemic autopsy and toxicological screening, the so-called sudden unexplained death (SUD). Fatal arrhythmia may be a chief cause of the SUD. In our patients, two cases (12.67%) were not revealed of positive pathological changes after systematically autopsy. Studies have documented that SUD is a significant contributor to the increased mortality seen in schizophrenia, with an estimated rate for up to 20% mortality (30). The unexplained deaths were found at all ages, ranging from 2 to 86 years old. Male decedents are more often observed, and dyslipidemia and diabetes are more common in these patients (31). The exact pathophysiological mechanism of SUD remains poorly understood, but it is likely that fatal cardiac arrhythmia may play essential roles. Administration of antipsychotics, especially second-generation antipsychotics, may prolong QT interval and trigger severe ventricular arrhythmias, including *torsades de pointes*, and thereby resulting in unexplained death (32). It was reported that use of Risperidone even at a low dose in an apparently healthy individual is associated with increased risk of lethal ventricular tachyarrhythmia (33). The SUD cases in the present study had no history or clinical symptoms of cardiac diseases and were treated with Risperidone at 4 and 2 mg daily, respectively, during hospitalization, all at a therapeutic dose. Therefore, from the clinical perspective, real-time ECG monitoring prior to and after starting such treatment is very important to avoid such fatal outcomes. In forensic practice, in cases where autopsies have not reported structural cardiac abnormalities, death may be due to an underlying primary arrhythmogenic disease such as long or short QT syndrome, Brugada Syndrome, and catecholaminergic polymorphic ventricular tachycardia, the postmortem genetic examinations (molecular autopsy) would provide a possible solution to determine the cause of death (5, 32). Therefore, to better understand the genetics and their relation with morbidity and mortality of SCD or SUD in schizophrenia, genetic screening was suggested to be implemented. While standard testing approaches such as which genes and mutation types should be detected have not yet been established, future genetic studies are necessary in the fields of forensic practice and in clinical application.

Our study was limited by the small number of patients involved. Also, due to the technical limitations, we were unable to obtain sufficient information of theses patients' drug regimens and heart function data before the last hospitalization. Collaborative studies involving both clinical physicians and forensic pathologists would be more helpful in illustrating the characteristics of SCD in schizophrenia.

## Conclusion

In all, we presented nine autopsy-based cases and reported both clinical and autopsy characteristics of these schizophrenia decedents during hospitalization. The SCD cases comprised of both male and female patients and these patients died within 15 days after hospitalization. The causes of death were mostly related to ischemic or structural heart diseases, while the cause of death for a small portion (two cases) remained unexplained. Postmortem autopsy also identified hepatic stenosis and respiratory inflammation as the most common associate extra-cardiac lesions. Critical attention should be paid to the risk of adverse effect of antipsychotics for the schizophrenia patients, and a routine ECG monitor should be a priority for the psychiatrists to avoid fatal outcomes.

## Data Availability Statement

The original contributions presented in the study are included in the article/supplementary materials, further inquiries can be directed to the corresponding author.

## Ethics Statement

The studies involving human participants were reviewed and approved by the Ethical Review Board at the School of Basic Medical Science, Gannan Medical University (Approval No.: 2021-217). The patients/participants provided their written informed consent to participate in this study.

## Author Contributions

YC analyzed data and wrote the original draft. FZ, YY, SW, and LZ were involved in the clinical and forensic data collection. FY conceived and designed the study and edited the manuscript. All authors have read and agreed to the published version of the manuscript.

## Funding

This work was financially funded by the Open Project of Key Laboratory of Forensic Pathology, Ministry of Public Security (No. GAFYBL201903), the Science and Technology Project of Jiangxi Provincial Department of Education (No. GJJ160979), the Research Project of Gannan Medical University (No. XN201924), and Open Project of Key Laboratory of Prevention and Treatment of Cardiovascular and Cerebrovascular Diseases of Ministry of Education (No. XN201810).

## Conflict of Interest

The authors declare that the research was conducted in the absence of any commercial or financial relationships that could be construed as a potential conflict of interest.

## Publisher's Note

All claims expressed in this article are solely those of the authors and do not necessarily represent those of their affiliated organizations, or those of the publisher, the editors and the reviewers. Any product that may be evaluated in this article, or claim that may be made by its manufacturer, is not guaranteed or endorsed by the publisher.
